# The impact of malaria during pregnancy on low birth weight in East-Africa: a topical review

**DOI:** 10.1186/s12936-021-03883-z

**Published:** 2021-08-24

**Authors:** Line Bakken, Per Ole Iversen

**Affiliations:** 1 Department of Nutrition, University of Oslo, Oslo, Norway; 2 Department of Blood Transfusion and Haematology, Muhimbili University of Health and Allied Sciences, Dar-es-Salaam, Tanzania

**Keywords:** East Africa, Low birth weight, Malaria, Pregnancy, Sulfadoxine-pyrimethamine

## Abstract

**Background:**

Globally, approximately 15% of all babies are born with low birth weight (< 2.5 kg) and ≥ 90% of them are born in low- and middle-income countries. Malaria infection in pregnancy remains a public health concern as it can affect both the mother and the newborn. Notably, it increases the risk of newborns with low birth weight. The World Health Organization (WHO) recommends intermittent preventive treatment with ≥ 3 doses of sulfadoxine-pyrimethamine (SP) during pregnancy in areas with moderate to high malaria transmission in Africa. The aim of this topical review is to give an overview of the impact of malaria infection during pregnancy on low birth weight, with focus on East Africa where malaria is endemic.

**Methods:**

Eleven studies were selected according to a predefined set of criteria.

**Results:**

Three studies showed a significant reduction in the prevalence of low birth weight with intermittent preventive treatment with SP, whereas four studies found no significant impact of such treatment on low birth weight. The number of SP doses and compliance to this treatment may in part explain these discrepancies. Pregnant women with frequent symptomatic malaria infection had significantly higher risk of placental malaria.

**Conclusion:**

The WHO recommendation of ≥ 3 doses of intermittent preventive treatment with SP during pregnancy seem effective in preventing low birth weight, but treatment compliance is a challenge. Malaria prophylaxis is important during pregnancy, especially in endemic areas of malaria, such as East Africa.

## Background

Malaria in pregnancy affects more than 25 million pregnant women every year, both in high and low malaria-endemic areas [1]. Pregnancy is a period of increased vulnerability, even for those living in malaria-endemic areas, who develop immunity against malaria [2]. Therefore, malaria infection (especially with *Plasmodium falciparum*) during pregnancy remains a major public health problem, especially in sub-Saharan Africa [3, 4].

There are several adverse outcomes associated with malaria, which affect both the mother and the newborn, including stillbirth, preterm birth, maternal and neonatal mortality, congenital malaria, maternal anaemia and low birth weight (LBW, i.e. birth weight < 2.5 kg) [2, 5]. Importantly, LBW ranks among the most commonly documented adverse birth outcomes. In 2019, there were an estimated 33 million pregnancies in 33 moderate-to-high malaria transmission countries in the World Health Organization (WHO) African Region [1]. Thirty-five percent of these pregnancies were exposed to malaria infection, and it is estimated that malaria infection during the pregnancy resulted in 822.000 infants with LBW. In line with this, Eisele et al*.* [6] estimated that 11% of neonatal deaths in sub-Saharan Africa are due to LBW associated with malaria infections during pregnancy. The WHO therefore recommends intermittent preventive treatment during pregnancy (IPTp) with sulfadoxine-pyrimethamine (SP) in all areas with high or moderate malaria transmission in Africa, and all women should receive least 3 doses of IPTp-SP during their pregnancy, each dose being given at ≥ 1 month apart, starting as early as possible in the second trimester [7].

According to a systematic review from 2019, 20.5 million infants worldwide were born with LBW, which represents 14.6% of all births. Of these LBW infants, about 90% were born in low- and middle-income countries (LMICs), and in sub-Saharan Africa the prevalence of LBW was 24% [1].

In pregnancy, *P. falciparum* tends to sequester on the placenta and thus the density of blood parasites available is below the detection limit of microscopy and, therefore, can only be detected by sensitive molecular tools, thus being a submicroscopic parasitaemia [8, 9]. Placental malaria infection is characterized by sequestration of malaria-parasite-infected erythrocytes and infiltration of immune cells in, as well as thickening of the placenta, which cause altered exchange of nutrients and waste products between mother and her fetus [10].

There is a paucity in compiling data from studies of malaria and LBW in East Africa, a part of sub-Saharan Africa endemic for falciparum malaria. Such information might be important, not only for the pregnant woman herself and the antenatal care staff, but also for other stake-holders and policy-makers. To address this knowledge gap, provide a topical review is here provided of the relationship between malaria during pregnancy and LBW with focus on East-Africa.

## Literature search-strategy

In this topical review a list of relevant MeSH words was generated and used to search for relevant articles from the database PubMed. The search was performed on 25 January, 2021. The search covered research conducted in East African countries, which according to PubMed consists of Burundi, Djibouti, Eritrea, Ethiopia, Kenya, Rwanda, Somalia, South Sudan, Sudan, Tanzania and Uganda. The search covered peer-reviewed research published between January 2000 and January 2021, and the articles from PubMed were imported into an EndNote library. Search terms included “Infant, Low Birth Weight”, “Malaria”, and “Africa, Eastern”. Studies were included in this review if they focused on (i) infants with LBW; (ii) malaria infection in pregnancy; (iii) were conducted in East Africa, and (iv) included an abstract and the free full text in English was available. Reviews or meta-analysis were not included in the selected literature-search, however we included such reports if relevant in our discussion. The inclusion of studies were not restricted to any particular study design, hence e.g. randomized trials and observational studies were eligible for inclusion. Studies that did not examine associations between infants with LBW and malaria infection during pregnancy in East Africa, were excluded. Studies that did not have LBW as a primary outcome were also excluded.

## Results

**Fig. 1:**
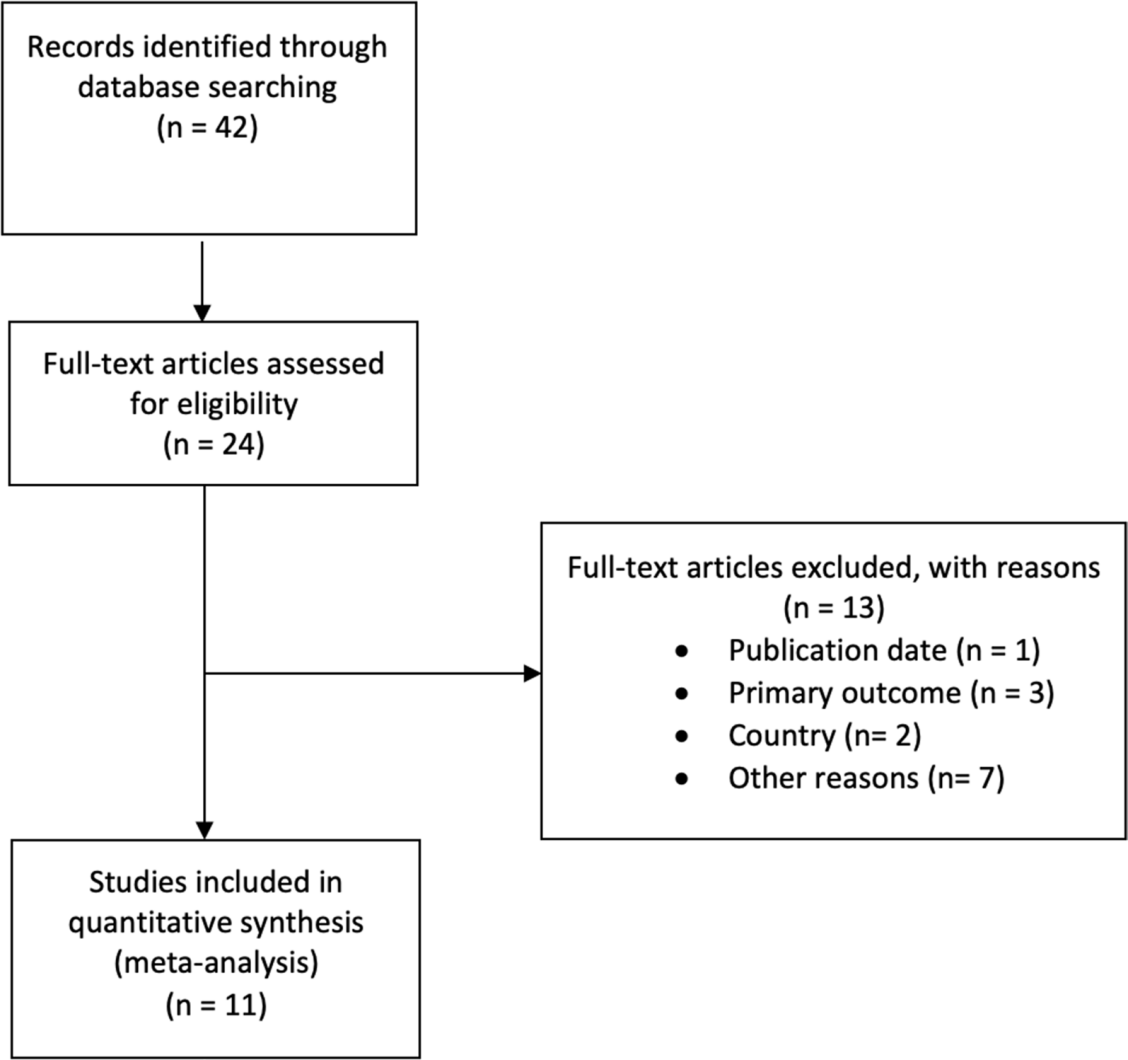
Flow chart showing the selection of articles included in this topical review

The literature search initially retrieved 42 articles (Fig. 1). Taking into account the exclusion criteria listed above, we were left with 11 articles summarized in Tables 1 and 2.

**Table 1:** Summary table of studies examining the effect of malaria treatment during pregnancy on low birth weight infants

Authors	Country	Study design (year)	Sample size	Treatment; frequency	Method for determining malaria infection	Results/comment
Braun et al. [11]	Western Uganda	Cross-sectional study (2013)	915	IPTp, SP; Start in 2nd trimester, then with every ANC visit	Polymerase chain reaction of parasite DNA	*P. falciparum*^a^ infection was significantly associated with LBW^b^ IPTp^c^—> no significant influence on the presence of *P. falciparum* infection
Mbonye et al. [12]	Mukono district, Uganda	Non-randomized community trial (2003–2005)	2785	IPTp, SP; One dose in 2nd and one in 3rd trimester	Self-reported (based on fever, headache, joint pain, general weakness)	Lower prevalence of LBW (6%) with the new delivery system vs. with health units (p < 0.03)
Mikomangwa et al. [13]	Dar-es-Salaam, Tanzania	Facility-based observational cross-sectional study (2018)	631	IPTp, SP; Start in 2nd trimester, then with every ANC visit	Rapid antigen-based test	The prevalence of LBW was 6.5%. Malaria positive women had 11 times increased risk of LBW compared to those who were negative (p = 0.04) ≥ 3 doses of IPTp-SP^d^—> 83% decreased risk of LBW compared to those who did not use IPTp-SP (p = 0.05)
Mosha et al. [14]	Moshi and Rufiji, Tanzania	Prospective observational study (2012)	350	IPTp; Start in 2nd trimester, then monthly	Polymerase chain reaction of parasite DNA	No significant association between IPTp use and reduced risk of LBW
Ndeserua et al. [15]	Rufiji, Tanzania	Cross-sectional study (2012)	350	2 doses SP; One dose in 2nd and one in 3rd trimester	Quantification of parasites in blood smear	Two doses SP during pregnancy was insignificantly associated with risk of LBW (p = 0.73)
Ndyomugyenyi et al. [16]	Kabale, Uganda	Randomized controlled trial (2004–2007)	5775	ITN^e^ + placebo or ITN + IPTp or IPTp; Start in 2nd trimester, then with every ANC visit	Quantification of parasites in blood smear	There was no significant difference between the three intervention groups in the prevalence of LBW (p = 0.802)
Van Eijk et al. [17]	Kenya	Cohort study (1999–2000)	889	IPTp, SP; Start in 2nd trimester, then with every ANC visit	Quantification of parasites in blood smear	1 dose IPTp—> associated with a mean increased BW^f^ of 54 g (p = 0.11) ≥ 2 doses IPTp- > associated with a mean increased BW of 128 g (p = 0.004) compared with mothers who had not used IPTp

^a^Plasmodium falciparum, ^b^Low birth weight, ^c^Intermittent preventive treatment during pregnancy, ^d^Intermittent preventive treatment during pregnancy with sulfadoxine-pyrimethamine, ^e^Insecticide-treated nets, ^f^Birth weight. Note, all papers used the WHO definition of LBW (birth weight < 2.5 kg)

**Table 2:** Summary table of studies examining placental malaria and low birth weight infants

Authors	Country	Study design (year)	Sample size	Treatment; frequency	Method for determining malaria infection	Results/comment
Kalinjuma et al. [18]	Dar-es-Salaam, Tanzania	Cohort study (2010–2013)	1115	SP^a^; Start in 2nd trimester, then with every ANC visit	Polymerase chain reaction of parasite DNA; quantification of parasites in blood smear; histology of placental tissue	PM^b^ was not significantly associated with LBW^c^
Kapisi et al. [4]	Tororo, Uganda	Cohort study (2014)	282	SP; Start in 2nd trimester, then given every 8th week	Quantification of parasites in blood smear; histology of placental tissue	Malaria burden during pregnancy—> PM—> risk of LBW (trends, not significant)
Mohammed et al. [19]	Central Sudan	Case–control study (2010)	174	No information given	Polymerase chain reaction of parasite DNA; quantification of parasites in blood smear; histology of placental tissue	Submicroscopic malaria infection during pregnancy—> significantly higher risk of having a LBW delivery
Dong et al. [20]	Tanzania	Cohort study (2002–2005)	882	No information given	Quantification of parasites in blood- and placental smear	CXCL9^d^ was significantly associated with LBW among malaria-infected primigravidae

^a^Sulfadoxine-pyrimethamine, ^b^Placental malaria, ^c^Low birth weight, ^d^CXC ligand 9. Note, all papers used the WHO definition of LBW (birth weight < 2.5 kg)

### Intermittent preventive treatment with sulfadoxine-pyrimethamine in pregnancy on prevalence of low birth weight

Table 1 summarizes the studies that examined the effect of intermittent preventive treatment during pregnancy with IPTp-SP on birth weight and LBW in particular.

Braun et al*.* [11] did a cross-sectional study among 915 delivering women in Western Uganda to examine the association between IPTp-SP and malaria infection, maternal anaemia, LBW and preterm delivery. At the time this study was conducted, the Ugandan policy recommendation was to have at least two doses of IPTp-SP during pregnancy. Malaria infection was significantly associated with LBW. Moreover, more than 50% of the women had taken two doses, and about 80% had taken at least one dose of SP. Importantly, this treatment had no significant impact on the presence of malaria infection compared with women who did not take SP. They found a non-significant reduction in anaemia, LBW and preterm delivery, and thus concluded that IPTp-SP did not provide an observable benefit.

In a non-randomized community trial in Uganda, Mbonye et al*.* [12] investigated two doses of IPTp-SP on malaria during pregnancy. This study assessed a new delivery system and its effect on maternal health and pregnancy outcomes. The intervention group received IPTp-SP through community resource people (the new delivery system), whereas the control group received IPTp-SP at health units. The intervention group registered a significantly lower prevalence of LBW (6%) compared with the control group (8.3%).

Mikomangwa et al*.* [13] did a facility-based observational cross-sectional study in Dar-es- Salaam, Tanzania. They investigated adverse birth outcomes among 631 pregnant women who received IPTp-SP in a low malaria transmission region. Women who used at least one dose of IPTp-SP had a prevalence of malaria at 0.6% and prevalence of LBW of 6.5%. Women with malaria infection had 11 times increased risk of LBW compared to those who did not have malaria infection (p = 0.04). Women who used ≥ 3 doses of IPTp-SP had 83% decreased risk of LBW compared to those who did not use IPTp-SP (p = 0.05).

Mosha et al*.* [14] investigated the effectiveness of IPTp-SP (one or two doses) during pregnancy on placental malaria, maternal anaemia and birth weight in areas with different malaria transmission intensity in Tanzania. IPTp-SP was associated with a significant decreased risk of placental malaria. This effect was only pronounced in areas with high malaria transmission (p = 0.02) and not in areas with low malaria transmission (p = 0.48). IPTp-SP use was not significantly associated with reduced risk of LBW.

In a hospital-based cross-sectional study in Rufiji, Tanzania, Ndeserua et al*.* [15] investigated risk factors for placental malaria and associated adverse pregnancy outcomes. Use of ≥ 2 doses of IPTp-SP during pregnancy was not associated with decreased risk of placental malaria) (p = 0.08) or LBW (p = 0.73). This study thus concluded that two doses of IPTp-SP are ineffective in preventing and treating placental malaria and adverse pregnancy outcomes.

Ndyomugyenyi et al*.* [16] investigated the efficacy of malaria prevention during pregnancy in an area of low and unstable malaria transmission in Kabale highlands, South-Western Uganda. The study was an individually randomized placebo-controlled trial, where they used two doses of IPTp-SP and insecticide-treated nets. They examined the effect of these two treatment options as well as a combination of the two, on LBW. The prevalence of LBW was 6.5%. There was no significant difference between the three study groups in the prevalence of LBW.

In a hospital-based study in Western Kenya, Van Eijk et al*.* [17] investigated the effectiveness of IPTp-SP for control of malaria in pregnancy. The prevalence of placental malaria was 13.8% and the prevalence of LBW was 12.2%. There was a trend towards decreasing prevalence of LBW with increasing number of IPTp-SP doses (p = 0.02). Compared with birth weight of infants of mothers who had not used intermittent preventive treatment, one dose of IPTp-SP was non-significantly associated with an adjusted mean increase in body weight of 54 g (p = 0.11). Two or more doses of IPT-SP were significantly associated with an adjusted mean birth weight increase of 128 g (p = 0.004). Moreover, the trend test showed a significant association between an adjusted mean increase in birth weight of 61 g for each increase in number of IPT-SP doses.

### Placental malaria and low birth weight

Table 2 summarizes the studies that examined the effect of placental malaria, malaria burden during pregnancy, and submicroscopic malaria infection.

Kalinjuma et al*.* [18] did a cohort-study in Dar-es-Salaam, Tanzania among HIV-negative pregnant women to identify factors associated with submicroscopic placental malaria and its association with LBW. Women who did not use bed nets, fumigation or mosquito coils, had higher risk (75% increased risk) of submicroscopic placental malaria (odds ratio = 1.75; p = 0.03) compared with those who used malaria prevention. This study found no significant association between placental malaria and LBW.

A case–control study by Mohammed et al*.* [19] investigated submicroscopic *P. falciparum* infection and LBW in Central Sudan, an area with unstable malaria transmission. Cases were women who had LBW deliveries and the controls were women without LBW deliveries. The prevalence of submicroscopic malaria infection was higher in the cases compared with the controls: 27.6% *versus* 7% (p < 0.001). By using multivariate analysis, this study showed that malaria infection in the placenta was not significantly associated with LBW, while submicroscopic *P. falciparum* infection (p = 0.001) or a combination of submicroscopic infections and histologically determined infections (p = 0.012), were significantly associated with LBW.

Kapisi et al. [4] did a cohort-study based on data from a randomized controlled trial of intermittent preventive treatment during pregnancy among 300 Ugandan participants. In the parental randomized controlled trial they investigated placental malaria and delivery outcomes. SP was given every 8 weeks versus dihydroartemisinin-piperaquine (DP) given every 8 weeks versus DP given every 4 weeks. The available 282 women in the cohort-study were divided into three groups to examine the associations between the frequency of malaria infection during pregnancy and placental malaria. These three study groups were defined as follows: (i) “none” = women who had no episodes of symptomatic malaria or asymptomatic malaria during pregnancy; (ii) “low” = women who had 0–1 episode of symptomatic malaria; and (iii) “high” = women who had ≥ 2 episodes of symptomatic malaria during pregnancy. This study concluded that higher malaria burden during pregnancy was associated with placental malaria, and placental malaria was associated with increased risk for adverse birth outcomes like LBW, but there was no significant association between placental malaria and risk of LBW.

Dong et al*.* [20] investigated whether CXC ligand 9 (CXCL9) response to malaria infection during pregnancy was associated with LBW infants. CXCL9 is a small cytokine that is significantly upregulated and negatively correlated with birth weight. CXCL9 also plays a role in inducing chemotaxis, cause extravasation and promote differentiation and multiplication of leukocytes. Placental malaria was associated with decreasing birth weight in all study groups (p < 0.001). They also found a significant association between CXCL9 and reduction in birth weight. Interestingly, CXCL9 was significantly associated with decreased birth weight in placenta malaria positive primigravidae, but not among infants born to placenta malaria positive secundigravidae or multigravidae.

## Discussion

Eleven original articles comprising 14,148 pregnant women were included in this topical review. Notably, with the current stringent search strategy, studies from only four of the 11 countries located in East Africa were retrieved. Seven studies investigated the effect of IPTp-SP [11–17], whereas the remaining four investigated adverse pregnancy outcomes associated with submicroscopic placental malaria [18, 19], infection with *P. falciparum* and measures of placental malaria [4] and CXCL9 response to malaria during pregnancy [20]. Only three studies found a significant reduction in the prevalence of LBW with IPTp-SP [12, 13, 17].

Kapisi et al*.* [4] divided the women into three groups and found that high malaria burden during pregnancy led to significantly increased risk of placental malaria compared with the other two groups with a lower malaria burden. Even though increasing malaria burden was significantly associated with placental malaria, there were trends, but not significant, of an association between placental malaria and LBW [4, 18, 19]. Another study found a significant association between placental malaria and LBW by investigating placental CXCL9 [20]. In contrast, the case–control study from Central Sudan showed that women who had LBW deliveries had significantly higher prevalence of submicroscopic malaria infection compared to those who did not have LBW deliveries [19]. In other words, it appears that placental malaria may not be significantly associated with LBW, but submicroscopic *P. falciparum* infection may [4, 19]. However, due to the limited evidence, a firm conclusion on this cannot be drawn.

Most studies in this review investigated the effect of one to three doses of IPTp-SP [11–17]. A systematic review and meta-analysis by Kayentao et al*.* [21] investigated intermittent preventive treatment for malaria in pregnancy using two versus three or more doses of SP and its association to LBW in Africa. The reason they chose to study the difference between two and three or more doses is that two doses may not provide protection during the last four to ten weeks of pregnancy, which is an essential period for fetal weight gain. They found that among pregnant women in sub-Saharan Africa, IPTp-SP with three or more doses was associated with a higher birth weight and a decreased risk of LBW compared with the standard 2-dose regimen. These data provide support for the recommendations from WHO to provide at least three doses of IPTp-SP [7, 21]. Mikomangwa et al*.* [13] found that ≥ 3 doses of IPTp-SP led to an 83% decreased risk of LBW compared to those who did not use IPTp-SP. These results support the study of Kayentao et al*.* [21].

A systematic review and meta-analysis from 2015 of randomized and quasi-randomized trials investigated anti-malarial drugs for preventing malaria during pregnancy and the risk of LBW [22]. This review found that all combined anti-malarial drugs were associated with a 27% decreased risk of LBW compared with no use. The authors concluded that SP may no longer protect against the risk of LBW in areas of high-level drug resistance in Africa. A recent meta-analysis investigated the effect of *P. falciparum* SP resistance on the effectiveness for malaria in pregnancy in Africa [23]. The results showed that IPTp-SP increased the prevalence of molecular markers of SP resistance and it was correlated with a decrease in the effectiveness of SP to prevent malaria infections and LBW. Based on these findings it was suggested that monitoring of SP resistance could be a policy tool to guide the use of IPTp-SP [23].

The study from Central Sudan found that among women who had LBW deliveries, the prevalence of submicroscopic malaria infection was significantly higher compared with the controls [19]. A limitation in this study is the low sample size. In line with this, the study failed to obtain enough submicroscopic malaria infections to enable parasite genotyping. Hence, a cross-sectional study with a larger sample size is needed. In areas with stable malaria transmission, fever during pregnancy has been reported as an important predictor of placental malaria, but Ndeserua et al*.* [15] could not differentiate between confirmed malarial illness and non-malarial fever during the pregnancy. The study had a small sample size as well, and as a result of that they only studied one district, so the findings cannot be generalized to the larger Tanzanian population.

There has been a significant progress in reducing the prevalence of *P.falciparum* over the past decade, especially in Africa. A review by Rijken et al*.* [24] investigated malaria in pregnancy in the Asia–Pacific region. They found that malaria in pregnancy in this region contrasts with that in Africa because many women are at risk in highly heterogeneous transmission settings. Most of the pregnant women had little or no background immunity to malaria, so each infection is potentially fatal to the women and their fetuses. Moreover, they found that the prevalence of reduced birth weight of newborns was similar to that recorded in Africa, but effects of symptomatic malaria during pregnancy seemed to be more prominent in the Asia–Pacific region compared to Africa. Most pregnant women who live in the Asia–Pacific region are at risk for infection with *Plasmodium vivax*, but prevention of malaria during pregnancy is not emphasized in national guidelines in the Asia–Pacific region as it is in sub-Saharan Africa. In line with this, the WHO recommendation for interventions for the control of malaria during pregnancy are mostly based on findings from sub-Saharan Africa. Based on this, several studies should examine malaria during pregnancy and make strategies to prevent malaria infection during the pregnancy for the Asia–Pacific region as well.

The aim was to summarize data collected during the past 20 years (i.e. in the period after the WHO recommended to use IPTp-SP) on the relationship between pregnancy and malaria with focus on LBW infants in Eastern Africa in the form of a topical review to provide and facilitate such information to relevant stake-holders (e.g. the pregnant women, health-staff and policy-makers). There are some limitation to this approach, e.g. there are several challenges in measuring the prevalence of LBW because many infants in LMICs are not weighed at birth, some are misclassified and the quality of data-reporting varies [5]. In addition, estimates of LBW in LMICs are mainly based on data compiled from health facilities, but these may be under-estimates because some newborns are delivered at home, in particular those of poor households [25]. Moreover, any heterogeneity among the selected studies was not quantified, or of any confounding factors. Furthermore, there might be differences among the studies regarding the diagnosis of malaria in pregnancy. The possible impact on seasonal variations, e.g. related to climate, was not included.

## Conclusion

This topical review reports that among pregnant women in sub-Saharan Africa, three or more doses IPTp-SP were associated with a decreased risk of LBW and an increased birth weight compared to the standard 2-dose regimen. These studies provide support for the current WHO recommendation to provide at least three doses of IPTp-SP during pregnancy. Some studies concluded that SP may no longer protect against the risk of LBW in those areas of high-level resistance in Africa. Future studies should thus monitor the SP-resistance to guide the use of IPTp-SP. Women who did not use mosquito prevention methods such as bed nets had higher risk of submicroscopic placental malaria. Based on these results, one should continue to advice pregnant women in East Africa (and probably also in other areas with endemic malaria) should use bed nets or other mosquito prevention methods to decrease the risk of submicroscopic malaria.

## Data Availability

Available on request.

## Abbreviations

LBW: Low birth weight
IPTp-SP: Intermittent preventive treatment-sulfadoxine-pyrimethamine
LMICs: Low- and middle-income countries
DP: Dihydroartemisinin-piperaquine
CXCL9: CXC ligand 9
